# Interplay between Redox Signaling, Oxidative Stress, and Unfolded Protein Response (UPR) in Pathogenesis of Human Diseases

**DOI:** 10.1155/2019/6949347

**Published:** 2019-04-07

**Authors:** Tomasz Poplawski, Dariusz Pytel, Jaroslaw Dziadek, Ireneusz Majsterek

**Affiliations:** ^1^Department of Molecular Biology, Faculty of Biology and Environmental Protection, University of Lodz, Lodz, Poland; ^2^Department of Biochemistry and Molecular Biology, Hollings Cancer Center, Medical University of South Carolina, Charleston, South Carolina, USA; ^3^Institute for Medical Biology, Polish Academy of Sciences, Lodz, Poland; ^4^Department of Clinical Chemistry and Biochemistry, Medical University of Lodz, Poland

Endoplasmic reticulum (ER) stress triggers complex adaptive or proapoptotic signaling defined as the unfolded protein response (UPR), involved in several pathophysiological processes. Protein misfolding in the ER triggers the activation of three homologous transmembrane protein kinases, Ire1, the PKR-like ER kinase (PERK), and the transmembrane transcription factor ATF6. Protein folding is highly redox-dependent; the relations between the generation of oxidative stress and ER stress have become very interesting fields for investigation. Evidence suggests that ROS production and oxidative stress are not only coincidental to ER stress but also integral UPR components. These components are triggered by distinct types of ER stressors and facilitate either proapoptotic or proadaptive UPR signaling. Thus, ROS generation can be upstream or downstream UPR targets. Pathways involved in unfolded protein response are important for normal cellular homeostasis and organismal development and may also play key roles in the pathogenesis of many diseases. This special issue intends to address the different aspects relating interaction between oxidative stress, UPR, and cellular redox capacity. We are grateful to the Editorial Board of Oxidative Medicine and Cellular Longevity, who devoted this special issue to UPR, and all authors for their contribution. Many manuscripts were submitted, and after a detailed peer review process, three high-quality research works and two reviews on topics of novelty were selected to be included in this special issue.

In the review by Y. Rellmann and R. Dreier, mouse models with specific changes in protein folding are described. The authors mainly focus on those mouse models with ER stress-related chondrodysplasia phenotypes. This is particularly important as UPR downregulate essential processes of the endochondral ossification including chondrocyte proliferation and differentiation and ECM protein synthesis. In consequence, autophagy and apoptosis are triggered and lengths of long bones are reduced. However, the detailed description of various mouse models with ER stress is particularly relevant for readers studying different aspects of the UPR. The authors review among others mice with a knockout of proteins involved in ER folding machinery, UPR signaling, degradation of aggregated proteins, and protein trafficking and secretion. These mouse models could be useful for the study of the molecular pathogenesis of various ER stress-related human diseases as well as to propose novel treatment schemes. One of the ER stress-related diseases is acute kidney injury. The accumulation of unfolded protein in ER in the kidney is observed in aging and during sepsis; however, the molecular background of kidney injury is different. The aged kidney has reduced UPR and elevated oxidative stress in comparison with a younger one. Along this line is the paper by X. Liu et al. The authors report the increased susceptibility of an aged kidney to ER stress with excessive reactive oxygen species level using a mouse model. This phenomenon is connected with the loss of PERK phosphorylation and XBP-1 mRNA splicing. They also compared the level of ER stress in an aged kidney with and without an antioxidant and found that the antioxidant prevented kidney function failure induced by severe ER stress. A similar protective function against ER stress-induced kidney injury showed methane-rich saline as reported by Y. Jia et al. They found that the ER stress in the kidney is a consequence of sepsis and it is connected with oxidative stress and the ER stress-related apoptosis. They measured histopathological damage, the levels of proinflammatory cytokines, and reactive oxygen species in kidney tissues and apoptosis ratio. Methane has anti-inflammatory and antiapoptotic properties and seems to be a good candidate for supportive treatment in sepsis; however, the molecular mechanisms of methane remain to be elucidated. 67% hydrogen and 33% oxygen mixture (H_2_-O_2_) work in a similar way. S. Zhao et al. reported that H_2_-O_2_ mixture inhalation could protect against the cardiac dysfunction and structural disorders induced by ER stress in rats with chronic intermittent hypoxia (CIH). CIH is a consequence of obstructive sleep apnea—a common breathing disorder in humans. The authors found that H_2_-O_2_ improved cardiac dysfunction and attenuated CIH-induced ER stress by reduction of oxidative stress and cardiac apoptosis. An ER stress-oriented therapy is also used in cancer treatment. In the review by A. Walczak et al., the role of the ER-induced UPR pathway and the efficacy of its inhibitors and inducers in the inhibition of tumor progression are discussed. Correct protein processing and folding are crucial to maintain tumor homeostasis. Endoplasmic reticulum stress is one of the leading factors that causes disturbances in these processes. It is induced by impaired function of the ER and accumulation of unfolded proteins. Induction of ER stress affects many molecular pathways that cause the unfolded protein response. This is the way in which cells can adapt to the new conditions, but when ER stress cannot be resolved, the UPR induces cell death. The molecular mechanisms of this double-edged sword process are involved in the transition of the UPR either in a cell protection mechanism or in apoptosis. However, this process remains poorly understood but seems to be crucial in the treatment of many diseases that are related to ER stress.

A. Walczak et al. discuss that the UPR involvement in the pathogenesis and progression of various types of cancer is presented. Since it is a very promising target for novel anticancer therapy, more and more new molecules are being tested. A significant amount of them is naturally occurring chemicals that are present also in plants. Due to the abundance of the compounds affecting UPR, A. Walczak et al. have summarized the literature review on tested modulators in various cancer cell lines. Thus, the UPR modulators are a promising hope for a personalized therapy for patients in whom chemotherapy or radiotherapy has failed. It can become an innovative way to fight several different types of cancer. The response to a given compound depends on the phenotype of tumor cells, the severity of the disease, and the chemotherapy used so far.

The understanding of the ER stress response, especially in the aspect of pathological consequences of UPR, has the potential to allow us to develop novel therapies and new diagnostic and prognostic markers for cancer. It is emphasized that further experiments and analyses should be carried out using a variety of compounds that have the ability to inhibit and induce the UPR pathway in different types of cancers. It could also be useful in the treatment of noncancerous diseases including neurodegenerative disorders, i.e., glaucoma and Alzheimer's disease.

